# Development and content validation of patient-reported outcomes tools for ulcerative colitis and Crohn’s disease in adults with moderate-to-severe disease

**DOI:** 10.1186/s12955-022-01975-1

**Published:** 2022-05-07

**Authors:** Arpita Nag, Beverly Romero

**Affiliations:** 1 Takeda Development Center Americas, Lexington, MA USA; 2 ICON Plc, Gaithersburg, MD USA

**Keywords:** Crohn’s disease, Health-related quality of life, Inflammatory bowel disease, Patient-reported outcomes, Ulcerative colitis

## Abstract

**Background:**

Ulcerative colitis (UC) and Crohn’s disease (CD) are associated with a range of symptoms that adversely affect health-related quality of life. This research aimed to develop and validate two patient-reported outcome (PRO) tools to assess signs and symptoms in patients with moderate-to-severe UC or CD.

**Methods:**

PRO-UC and PRO-CD Diaries were developed in accordance with US Food and Drug Administration (FDA) recommendations. Data were collected from concept elicitation interviews (in which patients described their symptoms and experience of the disease) and further refined through cognitive interviews (in which patients assessed the relevance and clarity of questions in the tools).

**Results:**

Interviews were conducted with 12 patients for each indication. Five symptoms (urgent bowel movements, abdominal pain, frequent bowel movements, bloody stools, diarrhea/watery stools) were reported by 83–100% of participants with UC and were included in the final 6-item PRO-UC Diary: stool frequency, rectal bleeding (2 items), diarrhea, rectal urgency, and abdominal pain. For CD, seven symptoms (abdominal pain, diarrhea/loose stools, urgent bowel movements, fatigue/tiredness/weakness, frequent bowel movements, bloody stools, nausea) were reported by 50–100% of participants. These, together with vomiting and incontinence (reported by 42% and 33% of participants, respectively), were included in the final 10-item PRO-CD Diary, covering abdominal pain (2 items), stool frequency, liquid/very soft stool frequency, rectal bleeding, rectal urgency, nausea, vomiting, bowel incontinence, and general well-being. Symptoms were consistently cited across both indications to have an impact on quality of life, with frequent complaints being the need to always be near a toilet and inability to leave home, as well as general pain, discomfort, and nausea. For both tools, questions were accurately interpreted, with at least 67% of participants in both indications stating that items were easy to answer/relevant to their condition and symptoms were easy to recall over the last 24 h.

**Conclusions:**

Both the PRO-UC and PRO-CD Diaries were developed and validated in accordance with FDA recommendations, providing two new tools for use in clinical trials to assess response to treatment in patients with UC or CD. Psychometric analyses are warranted to fully evaluate their properties and value for use in clinical trials.

**Supplementary Information:**

The online version contains supplementary material available at 10.1186/s12955-022-01975-1.

## Background

Inflammatory bowel disease (IBD) is a chronic inflammatory disorder encompassing ulcerative colitis (UC) and Crohn’s disease (CD) [1, 2]. UC principally affects the colon; common complaints include rectal bleeding/mucus discharge, frequent stools, and lower abdominal pain [3], with symptoms usually peaking during early adulthood [4]. Most patients experience mild-to-moderate symptoms in a relapsing–remitting cycle; however, approximately 15% of patients experience more severe disease and, of these, 20% may need to be hospitalized owing to symptoms [5]. In contrast to UC, CD can affect the entire gastrointestinal tract, most commonly causing pain and chronic diarrhea (sometimes with gross bleeding), with other symptoms including fatigue, weight loss, and abdominal pain [6]. CD is principally a progressive disease, with just 20–30% of patients experiencing a non-progressive or indolent disease course [7]. Compared with healthy individuals, patients with UC and CD experience lower health-related quality of life (HRQoL) across several domains including psychological, physical, sexual and social aspects of life [8, 9]. In particular, active disease is associated with significant impairments in HRQoL [10, 11]; however, therapeutic interventions, including biologics (e.g., infliximab) and JAK inhibitors, have shown promise in improving HRQoL in individuals with IBD [8, 12].

Response to therapy, including the need for treatment intensification, is evaluated through a combination of monitoring of signs and symptoms and objective disease assessment by endoscopy, imaging, or measuring biomarkers of mucosal inflammation. Accurate assessment of symptoms and response to therapy are particularly important in the clinical trial setting, especially in the context of providing evidence to support label claims.

Patient-reported outcomes (PROs) are increasingly used in clinical trials alongside conventional clinical endpoints to quantify the patient perspective in terms of how they feel and function, and are recommended by both the European Medicines Agency (EMA) and the US Food and Drug Administration (FDA) [13, 14]. They are designed to assess the impact of a disease or treatment on overall functioning, HRQoL, and symptoms. Additionally, PRO measures are considered an important tool for supporting PRO claims in approved product labeling, as well as demonstrating product value to physicians, patients, and payers. FDA guidance specifically recommends that development of PRO tools should involve qualitative research within the target population to determine aspects of disease that most affect patients, and that findings should be embodied in the questions that make up the tool, which should then undergo psychometric assessment to test reliability and validity [14].

At the time of designing the studies described herein, there were no PRO tools for assessment of UC or CD that met the FDA criteria, although two tools, the Ulcerative Colitis Patient-Reported Outcomes Signs and Symptoms (UC-PRO/SS [15]) and the Crohn’s Disease Patient-Reported Outcomes Signs and Symptoms (CD-PRO/SS [16]) measures have been developed in the interim. The purpose of the two studies described here was, therefore, to develop and validate the content of two additional patient diaries—the PRO-UC and PRO-CD Diaries—for assessing signs and symptoms in adults with moderate-to-severe disease, that would be suitable for use in clinical trials of new therapies for these two diseases and would meet the criteria defined by the FDA and the EMA.

## Methods

### Development of the initial versions of the tools

The initial version of the UC tool was adapted from the pediatric Daily Ulcerative Colitis Sign and Symptom Scale Diary [17]. Development was also informed by findings from an earlier UC concept elicitation study in adults [18] and regulatory guidance related to the Mayo stool frequency and rectal bleeding items [19, 20]. The initial version of the UC tool consisted of 6 items (absolute stool frequency, rectal bleeding [2 items: Mayo rectal bleeding item and absolute frequency of stools with blood], absolute frequency of loose/watery stools, absolute frequency of stools with urgency, and worst abdominal pain rated on a numeric scale ranging from 0 to 10), each with a 24-h recall period completed daily by patients.

The initial version of the CD tool consisted of 11 items, each with a 24-h recall period, which patients completed daily. Abdominal pain was assessed over 2 items, with the remaining items assessing stool frequency, liquid/very soft stool frequency, rectal bleeding, stools with blood, rectal urgency, nausea severity, vomiting, bowel incontinence, and general well-being. Abdominal pain was assessed using an 11-point numeric rating scale and a 4-point verbal rating scale; nausea severity was rated using a 4-point scale from none to severe; general well-being was rated on a 5-point scale from terrible to generally well; the remaining items required participants to enter a numerical value for the past 24 h.

Refinement of the initial tool content and subsequent content validation of the revised tool were assessed via one-on-one, semi-structured, qualitative interviews with consenting participants, as described below.

### Participant interviews

Participants were recruited and screened through a US market research recruitment agency, Global Perspectives. Individuals in the Global Perspectives database (consisting of patients with a wide variety of medical conditions who had all given prior permission to be contacted for research purposes) who matched the inclusion/exclusion criteria for the study were invited to take part.

Participants considered for the study were male or female, aged 18–80 years, had a diagnosis of UC or CD (either active or in remission; those with unspecified or unclassified IBD were excluded), and were willing/able to participate in a 60–90-min interview. Only participants with moderate-to-severe UC or CD were included, as defined by: having a self-reported score of 3–5 on a scale ranging from 1 (normal) to 5 (very severe) and a physician-reported score of 3–4 on a scale ranging from 1 (normal) to 4 (severe); having seen a physician within the last 6 months; and having the diagnosis confirmed by submitting a physician-completed form or a photograph of a current UC/CD prescription. Participants with CD were additionally required to be receiving a biologic treatment for CD. Participants who had received an ileostomy with either a subtotal colectomy or a proctocolectomy were excluded, as were those with UC who had received a restorative colectomy with anal pouch, and those with CD who had received stricturoplasty, any type of colectomy/bowel resection, or proctocolectomy. Participants with a previous diagnosis of irritable bowel syndrome were also excluded.

Participant interviews included concept elicitation (participants were asked to describe symptoms and their experience of the disease) and cognitive interviewing methodology to assess the relevance, clarity, and appropriateness of the PRO tools. Interviews were conducted in three rounds. The first two rounds were conducted over the telephone using paper-based administration of the tool; questions were modified after each round. The third round was delivered in an electronic format, the e-diary, and was assessed in face-to-face interviews. All data collection and recruitment procedures met institutional review board (IRB) requirements, with study protocols approved by a central IRB, Salus IRB. Written informed consent was obtained from study participants prior to completing any study-related activities.

COREQ (COnsolidated criteria for REporting Qualitative research) item responses for the qualitative interviews that contributed to early development of the PRO-UC and PRO-CD Diaries are presented in Additional file 1 [21].

### Analysis

All interviews were audio-recorded and transcribed verbatim. Data for the PRO-UC and PRO-CD Diaries were analyzed separately using a combination of thematic and content analysis [22]. De-identified transcripts were analyzed using MaxQDA v11 qualitative analysis software (VERBI GmbH), with preliminary analyses from the first few interviews conducted to ensure that the interview guide was generating sufficient patient descriptions of UC/CD signs and symptoms. Participant demographics and characteristics were summarized using descriptive statistics.

## Results

### Study participants

For each indication—UC and CD—12 participants were included, with four participants interviewed in each of three rounds. Participants were aged 31–59 years in the UC study and 18–61 years in the CD study, and 75% of participants in both studies were in remission at the time of the interview (Table 1). In both groups, 10 participants (83%) and two participants (17%) were receiving anti-tumor necrosis factor therapy and anti-integrin therapy, respectively. Five participants (42%) and two participants (17%) in the UC and CD groups, respectively, were receiving 5-aminosalicylic acid treatment. During the interview, it was discovered that one participant with UC had received a colectomy; data for this participant were retained as the participant was able to describe their experience pre- and post-surgery. Five participants (42%) in each study reported comorbidities.

**Table 1:** Participant characteristics

	UC (N = 12)	CD (N = 12)
Age, mean (range), years	45 (31–59)	43 (18–61)
Gender, n (%)		
Male	8 (67)	3 (25)
Female	4 (33)	9 (75)
Race, n (%)		
Caucasian	9 (75)	6 (50)
African American	1 (8)	4 (33)
Hispanic	1 (8)	0 (0)
Mixed race	1 (8)	2 (17)
Work status, n (%)		
Full-time	12 (100)	8 (67)
Part-time	0 (0)	1 (8)
Unemployed	0 (0)	2 (17)
Unable to work	0 (0)	1 (8)
Current treatment status, n (%)		
Remission	9 (75)	9 (75)
Flare	3 (25)	3 (25)
Disease-specific treatment, n (%)^a^		
Anti-TNF	10 (83)	10 (83)
5-ASA	5 (42)	2 (17)
Anti-integrin	2 (17)	2 (17)
Other^b^	8 (67)	11 (92)
Time since diagnosis, mean (range), months	103 (19–478)	103 (19–478)
Time since last flare, mean (range), months	8 (3–20)	8 (3–20)
Any other medical conditions, n (%)	5 (42)^c^	5 (42)^d^

ASA, aminosalicylic acid; CD, Crohn’s disease; TNF, tumor necrosis factor; UC, ulcerative colitis

^a^Participants could be receiving more than one therapy

^b^Other treatments included azathioprine, balsalazide, corticosteroid, lorazepam, methotrexate, mercaptopurine, and probiotics

^c^Comorbidities reported by patients with UC: ankylosing spondylitis, asthma, gastroesophageal reflux disease, hypothyroidism, and type 2 diabetes (n = 1 for each)

^d^Comorbidities reported by patients with CD: high cholesterol and high blood pressure (n = 1); polycystic ovary syndrome and Wegener's granulomatosis (n = 1); Tourette’s syndrome (n = 1); cellulitis, asthma, and osteoarthritis (n = 1); and benign prostate condition, depression, back pain, and incontinence (n = 1)

### Ulcerative colitis

#### Concept elicitation

Participants reported experiencing 24 different signs and symptoms during the course of their disease (Table 2). The most commonly reported symptoms (reported by ≥ 50% of participants) were urgent bowel movements (n = 12), abdominal pain (n = 11), frequent bowel movements (n = 11), bloody stools (n = 10), diarrhea/watery stools (n = 10), and nausea (n = 6). Except for nausea, these symptoms were also spontaneously reported by at least 50% of participants experiencing the reported symptom.

**Table 2:** Symptoms reported by at least four participants in the UC study (N = 12)^a^

Symptom^b^	Total reported^c^	Spontaneous report^d^	Probed report^d^
Urgent bowel movements^e^	12 (100)	6 (50)	6 (50)
Abdominal pain^e^	11 (92)	10 (91)	1 (9)
Frequent bowel movements^e^	11 (92)	9 (82)	2 (18)
Bloody stools^e^	10 (83)	8 (80)	2 (20)
Diarrhea/watery stools^e^	10 (83)	9 (90)	1 (10)
Nausea	6 (50)	2 (33)	4 (67)
Fatigue	5 (42)	4 (80)	1 (20)
Lack of appetite	4 (33)	4 (100)	0 (0)

PRO, patient-reported outcomes; UC, ulcerative colitis

^a^Symptoms reported spontaneously by 1–3 participants: bloating (n = 3), joint pain (n = 2), vomiting (n = 2), constipation (n = 1), dehydration (n = 1), difficulty swallowing (n = 1), dizziness (n = 1), fever (n = 1), excessive gas (n = 1), general discomfort (n = 1), lower back pain (n = 1), malaise (n = 1), mouth sores (n = 1), mucus in stools (n = 1), skin discoloration (n = 1), and abdominal spasms (n = 1)

^b^Participants reported on current and past symptoms experienced

^c^Data are number of participants (%); percentage calculated as a proportion of the overall group (N = 12)

^d^Data are number of participants (%); percentage calculated as a proportion of the ‘total reported’

^e^Items in the PRO-UC Diary

**Fig. 1:**
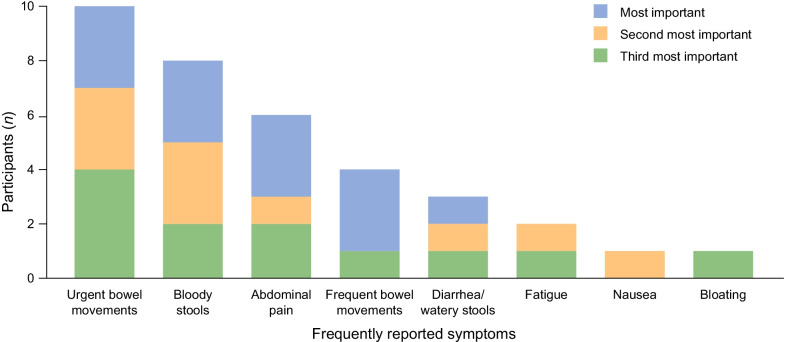
Relative importance of symptoms, as ranked by participants in the UC study (n = 11). UC, ulcerative colitis

Eleven participants ranked the relative importance of their symptoms (Fig. 1). Those most frequently reported in the top three for importance were urgent bowel movements (n = 10), bloody stools (n = 8), abdominal pain (n = 6), frequent bowel movements (n = 4), and diarrhea/watery stools (n = 3), all of which were considered the most important symptom by at least one participant. Concept saturation was assessed based on the number of interviews taken to identify the symptoms, with 75% (18/24) being identified within the first nine interviews, 92% (22/24) being identified by the tenth interview, and all 24 being identified by the twelfth interview.

Table 3 summarizes the terms used by participants to describe the six main symptoms during a UC flare and their reported impact on daily life. Frequently cited complaints across symptoms included an inability to leave home, work, or do normal activities, the need to always be near a bathroom, as well as general discomfort, nausea, and psychological impacts. Participants were also asked whether they experienced the symptoms during remission. Diarrhea/watery stools was the most frequently reported symptom in remission (n = 8) followed by frequent bowel movements (n = 7); bloody stools (n = 5), abdominal pain (n = 4), and urgent bowel movements (n = 3) were also reported by those in remission.

**Table 3:** Descriptions of symptoms during a UC flare reported in at least 50% of participants

Symptom	Descriptive terms	Impact on daily life
Urgent bowel movements	“Loss of control”, “need to go”, “sudden urge”, “urge to find bathroom in 10 to 15 s”, and “urgency to go to bathroom”	Inability to work; feeling that life was limited; being hospitalized; social impacts; limitations on clothing; feeling uncomfortable; dietary restrictions; and always needing to be near a bathroom
Abdominal pain	Pain described as “cramping/cramps”, “severe abdominal pain/abdominal pain”, “not a lot of pain”, and “stomach pain”	Inability to work, do usual activities, leave home, or go to school/sports practice; being cranky/not social; dietary restriction; difficulty sleeping; and psychological impacts
Frequent bowel movements	“10–15 times a day”, “12–25 times a day”, “15–20 times a day”, “30 times a day”, “a lot more often than normal”, “going to the restroom more frequently”, and “when it’s urgent, it’s frequent”	Inability to leave home; needing to be near a bathroom; inability to eat; and having to wake up early to let body calm down
Bloody stools	“Bleeding”, “blood in stools”, “rectal bleeding”, and “blood and mucus mixed together”	Psychological impacts; inability to leave home; feeling sick and nauseated; being unable to sit in a chair; and it is life-consuming
Diarrhea/watery stools	“Diarrhea”, “completely water”, “constant diarrhea”, “chronic diarrhea”, and “prolonged periods of diarrhea”	Feeling uncomfortable; inability to leave home; and being scared to go anywhere
Nausea	“Nausea” and “nausea all the time”	Not wanting to do anything and always having to carry mints

UC, ulcerative colitis

Good agreement was observed between the initial version of the tool and the symptoms identified through concept elicitation; all five of the symptoms reported most frequently by participants were included in the initial version of the PRO-UC Diary. The only other symptom reported in at least 50% of participants was nausea. However, this was reported spontaneously in only two participants, and only one of those reported it to be among the three most important symptoms. As a result, nausea was not added to the PRO-UC Diary.

#### Assessment of relevance and clarity of questions

During the cognitive interviews, participants described the relevance, clarity, and appropriateness of the questions comprising the draft version of the PRO-UC Diary. Most participants (67–100%) responded that each item was easy to answer and the symptoms easy to recall over the past 24 h (Table 4). All respondents accurately interpreted the questions and all but one respondent rated questions as being relevant (i.e. rating of ≥ 3 on a scale of 0–5), with at least half of the participants rating each item as extremely relevant (i.e. rating of 5). Four participants were asked about usability of the electronic device, and all reported the e-diary to be easy to use and complete.

**Table 4:** Results from cognitive interviews in the UC study (N = 12)

PRO-UC Diary item	Easy to answer	Symptoms easy to recall^a^	Accurate interpretation of question	Relevance^b^ ≥ 3	Relevance^b^ = 5
Rectal bleeding	12	11	12	12	6
*Rate your worst experience of rectal bleeding*					
Stool frequency	8	8	12	12	10
*Enter number of bowel movements passed*					
Stools with blood	12	12	12	11	10
*Enter number of bowel movements with blood*					
Diarrhea	11	10	11	11^c^	7
*Enter number of loose or watery bowel movements*					
Rectal urgency	11^d^	11^d^	12	12	10
*Enter number of bowel movements with urgency*					
Abdominal pain	12	11	12	12	7
*Rate your worst abdominal pain*					

Data are number of participants

PRO, patient-reported outcomes; UC, ulcerative colitis

^a^Recollection of symptoms over the past 24 h

^b^Participants rated how relevant the items were on a 6-point scale (from 0 = not at all relevant to 5 = extremely relevant)

^c^One participant accidentally skipped this question

^d^Only 11 participants were asked about recall and answerability of this item

In the course of the study, minor modifications were made to the wording of the questions for the UC tool, but no changes were made to the items included in the initial version or the scoring of items. The resulting 6-item tool is described below.

#### Scoring of the PRO-UC Diary

The final PRO-UC Diary is an electronic daily UC sign and symptom diary consisting of 6 individual items, each with a 24-h recall period, that describe the key symptoms experienced by patients with moderate-to-severe UC: (1) stool frequency, rectal bleeding (2 items; [2] severity [Mayo rectal bleeding item] and [3] frequency), (4) diarrhea/watery stools frequency, (5) rectal urgency frequency, and (6) abdominal pain. A Total Signs and Symptoms score is derived based on all items, with the exception of the Mayo rectal bleeding item (item 2: assessment of rectal bleeding severity). Frequency scores from items 1 and 3–5 (i.e. stool frequency, rectal bleeding frequency, diarrhea/watery stools frequency, and rectal urgency over a 24-h period) are categorized as follows: 0–2 events = 0; 3–5 events = 2.5; 6–8 events = 5.0; 9–11 events = 7.5; at least 12 events = 10. Item 6 (abdominal pain) is used as a raw score with no conversion. The Total Signs and Symptoms score is then calculated by averaging the categorized scores for items 1 and 3–5 and the score for item 6 over the last 3 days of available data at each time point (days were not required to be consecutive). The tool therefore provides a score ranging from 0 to 10, with higher scores indicating greater severity of signs and symptoms. In addition, items 1 and 2 (stool frequency and Mayo rectal bleeding) can be used to calculate Mayo stool frequency and rectal bleeding subscores, respectively, and the other 4 items can be combined to give a summary score.

### Crohn’s disease

#### Concept elicitation

Participants reported experiencing 21 different signs and symptoms during the course of their disease (Table 5), the most common of which (reported in ≥ 50% of participants) were abdominal pain (n = 12), diarrhea/loose stools (n = 11), urgent bowel movements (n = 10), fatigue/tiredness/weakness (n = 7), frequent bowel movements (n = 7), bloody stools (n = 7), and nausea (n = 6). Four other symptoms were reported in at least one third of participants (vomiting, incontinence, constipation, and joint aches/body aches). All signs and symptoms, except for nausea, vomiting, and incontinence, were spontaneously reported by at least 50% of participants experiencing the reported symptom.

**Table 5:** Symptoms reported by at least four participants in the CD study (N = 12)^a^

Symptom^b^	Total reported^c^	Spontaneous report^d^	Probed report^d^
Abdominal pain^e^	12 (100)	12 (100)	0 (0)
Diarrhea/loose stools^e^	11 (92)	10 (91)	1 (9)
Urgent bowel movements^e^	10 (83)	5 (50)	5 (50)
Fatigue/tiredness/weakness	7 (58)	7 (100)	0 (0)
Frequent bowel movements^e^	7 (58)	6 (86)	1 (14)
Bloody stools^e^	7 (58)	4 (57)	3 (43)
Nausea^e^	6 (50)	1 (17)	5 (83)
Vomiting^e^	5 (42)	2 (40)	3 (60)
Incontinence^e^	4 (33)	0 (0)	4 (100)
Constipation	4 (33)	4 (100)	0 (0)
Joint aches/body aches	4 (33)	4 (100)	0 (0)

CD, Crohn’s disease; PRO, patient-reported outcomes

^a^Symptoms reported by 1–3 participants: eye problems (n = 3), bloating/gas (n = 3), bruising (n = 2), difficulty eating certain foods (n = 2), general pain (n = 2), lack of appetite (n = 2), chest pain (n = 1), gastroesophageal reflux (n = 1), hemorrhoids (n = 1), and skin issues (n = 1)

^b^Participants reported on current and past symptoms experienced

^c^Data are number of participants (%); percentage calculated as a proportion of the overall group (N = 12)

^d^Data are number of participants (%); percentage calculated as a proportion of the ‘total reported’

^e^Items in the PRO-CD Diary

**Fig. 2:**
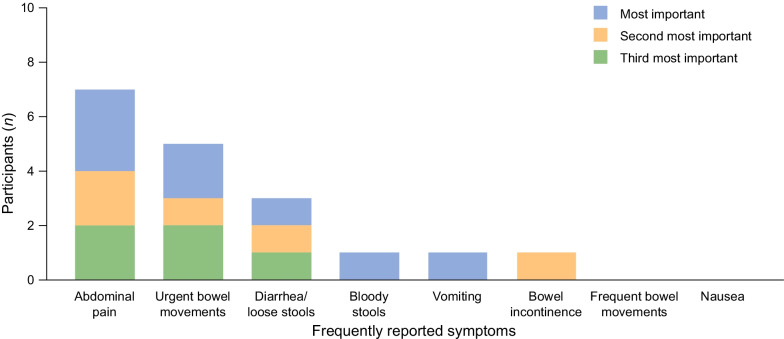
Relative importance of symptoms, as ranked by participants in the CD study (n = 11). CD, Crohn’s disease

Eleven participants ranked the relative importance of their symptoms (Fig. 2). Those most frequently reported in the top three for importance were abdominal pain (n = 7), urgent bowel movements (n = 5), and diarrhea/loose stools (n = 3), all of which were considered the most important symptom by at least one participant. Concept saturation was assessed based on the number of interviews taken to identify the symptoms, with 86% (18/21) being identified by the sixth interview and 100% of symptoms being identified by the eighth interview.

Table 6 summarizes the terms used by participants to describe the 11 most frequently reported symptoms during a CD flare and their reported impact on daily life. Themes reported consistently across symptoms included the inability to work or go out, the need to be near a toilet at all times, stress, and a negative impact on social life. Participants were also asked whether they experienced the symptoms during remission. Abdominal pain (n = 8) and diarrhea/watery stools (n = 8) were the symptoms most frequently reported by participants in remission; bloody stools (n = 6), frequent bowel movements (n = 6), urgency (n = 5), fatigue (n = 5), and constipation (n = 3) were also reported by those in remission. Joint/body aches (n = 2), nausea (n = 2), vomiting (n = 1), and incontinence (n = 1) were reported less frequently during remission.

**Table 6:** Descriptions of symptoms during a CD flare reported in at least 50% of participants

Symptom	Descriptive terms	Impact on daily life
Abdominal pain	“Abdominal pain”, “severe abdominal pain”, “tender when touched”, “abdominal/stomach aches and pain”, “severe cramping”, and “stepping on stomach with a cleat”	Inability to go to work; inability to concentrate or focus; and not wanting to go out
Diarrhea/loose stools	“Diarrhea/a lot of diarrhea”, “nothing formed”, “projectile”, and “uncontrollable diarrhea”	Afraid to/don’t leave house; affects sleep; embarrassing; have to wear Depends; and psychological impact
Urgent bowel movements	“Urgency”, “hard to control”, “more urgent to go”, “running to the bathroom”, and “urgency to evacuate”	Being stressful, mentally; having to be aware of surroundings; needing to be close to a bathroom; and always looking for a bathroom
Fatigue/tiredness/ weakness	“Chronic fatigue”, “lethargic”, “really tired”, and “weakness”	Difficulty doing anything and being unable to move
Frequent bowel movements	“10−12 times a day”, “20 times a day”, “continuously going to the bathroom”, and “frequent bowel movements”	Needing to be at home; being unable to go to work; affects social life; affects sleep; missing TV shows; being unable to work out; difficult to attend the movies; getting fired; and having to wear Depends
Bloody stools	“Bleeding”, “bloody stools”, “little blood in stool”, and “bleeding internally”	Feeling weak/anemic; having to stay close to home/bathroom; affects personal hygiene; have to be dependent on others; being unable to leave the house; and having no social life
Nausea	Nausea	Having to go to the ER and put life on hold; being unable to do much; needing to be home near a bathroom; being worried; and being late for work/missing work
Vomiting	“Throw up” and “vomiting”	Having to take time off from work; needing to be near home bathroom; being worried; having to stay home/can’t go out; and not being able to do daily tasks
Bowel incontinence	“If you’re nowhere near a bathroom but you have to go” and having “no place left to go and you need a bathroom and it’s not there”	Not asked
Constipation	“Straining”, “tough to go to the bathroom”, “constipated”, and “pain”	Everything is put on hold if hospitalized; and feeling bloated or gassy
Joint aches/body aches	“Joint pain”	Being hard to dance; having to use a walker; being unable to cook; and being unable to open a jar or bottle of water

CD, Crohn’s disease; ER, emergency room

Good agreement was observed between the initial version of the tool and the symptoms identified through the concept elicitation. All symptoms reported by at least half of the participants were included in the initial version of the PRO-CD Diary. Of the additional four symptoms (vomiting, incontinence, constipation, and joint aches/body aches) reported by at least one third of participants, vomiting and incontinence were included in the initial PRO-CD Diary as they were each cited by one patient as being one of the three most important symptoms, while vomiting was further described as having a substantial impact on daily living (e.g. participant having to take time off work, needing to stay at/near home, and not being able to do daily tasks). Constipation and joint aches/body aches were not added to the PRO-CD Diary as neither were included in the three most important symptoms reported by any participants. The resulting items included in the PRO-CD Diary were therefore abdominal pain (2 items), stool frequency, liquid/very soft stool frequency, rectal bleeding, rectal urgency, nausea, vomiting, bowel incontinence, and general well-being.

#### Assessment of relevance and clarity of questions

During the cognitive interview portion of the study, participants described the relevance, clarity, and appropriateness of the questions comprising the draft version of the PRO-CD Diary. Most participants (83–100%) found each item easy to answer, symptoms easy to recall over the last 24 h, and the questions easy to interpret (Table 7). At least seven participants rated every question as being relevant, except for the question related to vomiting, for which six participants (of 11 respondents) considered it to be relevant. Rectal bleeding was rated as relevant (score ≥ 3) by eight participants (67%) and considered extremely relevant (score = 5) by only six (50%). All five participants who were asked about usability of the electronic device found the e-diary easy to use and complete.

**Table 7:** Results from cognitive interviews in the CD study (N = 12)

PRO-CD Diary item	Easy to answer	Symptoms easy to recall^a^	Accurate interpretation of question	Relevance^b^ ≥ 3	Relevance^b^ = 5
Abdominal pain	11	12	12	11	9
*Rate your abdominal pain*					
Stool frequency	12	10	12	10	8
*Enter number of bowel movements passed*					
Liquid/very soft stool frequency	12	11	12	11	9
*Enter number of liquid or very soft stools*					
Rectal bleeding	12	11	12	8	8
*Enter number of bowel movements with blood*					
Rectal urgency	12	12	12	11	8
*Enter number of bowel movements with urgency*					
Worst abdominal pain	12	12	11	8^c^	7^c^
*Rate your worst abdominal pain*					
Nausea	11	12	12	7	6
*Rate your worst feeling of nausea*					
Vomiting	11	12	12	6^d^	6^d^
*Enter number of vomiting episodes*					
Bowel incontinence	12	12	10	7	6
*Enter number of incontinence episodes*					
General well-being	11	12	12	12	10
*Rate your general well-being*					
Worst rectal bleeding	11	12	12	8	6
*Rate your worst experience of rectal bleeding*					

Data are number of participants

CD, Crohn’s disease; PRO, patient-reported outcomes

^a^Recollection of symptoms over the past 24 h

^b^Participants rated how relevant the items were on a 6-point scale (from 0 = not at all relevant to 5 = extremely relevant)

^c^Only 10 participants were asked about relevancy of this item

^d^Only 11 participants rated the relevancy of this item

As with the PRO-UC Diary, in the course of the study, minor modifications were made to the wording of the questions for the CD tool. In addition, the item regarding worst experience of rectal bleeding was removed from the final tool as it received a relatively low rating for relevance. Furthermore, the response options for worst abdominal pain were changed from a 4-point response to an 11-point numeric rating scale.

#### Scoring of the PRO-CD Diary

The final PRO-CD Diary is an electronic daily CD sign and symptom diary consisting of 10 individual items that describe the key symptoms experienced by patients with moderate-to-severe CD: abdominal pain (2 items), stool frequency, liquid/very soft stool frequency, rectal bleeding frequency, rectal urgency frequency, nausea, vomiting frequency, bowel incontinence frequency, and general well-being. Several different scores can be calculated from the data collected, with the simplest output being individual scoring of items to assess the individual symptoms/key aspects of CD. In addition, several summary scores can be generated. As well as a Total Signs and Symptoms score calculated by averaging categorized/raw scores for individual items, the 11-point rating of abdominal pain can be combined with the general well-being score to calculate the CD Activity Index (CDAI) score, and the remaining items can be combined to produce a separate summary score. Alternatively, the 11-point rating of abdominal pain can be combined with the frequency of liquid/very soft stools to provide a 2-item summary score.

## Discussion

This study describes the development and validation of two PRO tools that can be used for assessing symptom burden and response to therapy in patients with UC or CD. The tools were developed following FDA guidance [14], which requires qualitative research performed among the target patient population, i.e. patients with moderate-to-severe UC or CD, as described in this paper. The semi-structured interviews employed in this study have enabled identification of the most relevant signs and symptoms for patients with UC and CD and ensured that the terminology used to describe these is easily understood by patients.

Concept elicitation identified six symptoms reported in at least half of the patients with UC, and seven symptoms reported in at least half of the patients with CD, with a further four symptoms being reported in at least one third of the patients with CD. Participants used a variety of terms to describe their symptoms, thus indicating the need for a PRO tool to provide consistent terminology to report such symptoms. The interviews with participants also revealed the impact of specific symptoms on daily activities, with participants using a variety of terms to describe the negative effects of symptoms on their quality of life.

The final tools, the PRO-UC Diary and PRO-CD Diary, provide concise means of assessing symptoms, based on responses to 6 items for the PRO-UC Diary and 10 items for the PRO-CD Diary, and should be valuable new tools for assessing baseline symptom severity and response to treatment. The resulting summary scores (Total Signs and Symptoms, Mayo rectal bleeding, and other symptoms for UC; Total Signs and Symptoms, CDAI, and the 2-item summary [abdominal pain and liquid/very soft stools] score for CD) provide quantitative measures of the most important symptoms from a patient’s perspective. These will allow comparison across patient groups and studies, including response to treatment.

Two alternative PRO tools have recently been developed for UC (the UC-PRO/SS [15]) and CD (the CD-PRO/SS [16]). The two UC tools cover similar aspects of the signs and symptoms of moderate-to-severe UC; the PRO-UC Diary additionally includes rectal bleeding, while the UC-PRO/SS additionally includes mucus in bowel movement, leak before reaching toilet, passing gas, and bloating in belly. Scores for individual items in the PRO-UC Diary are reported frequencies of symptoms, except for the assessment of abdominal pain. In contrast, all responses for the UC-PRO/SS are within a specified range of 0–4, except for the number of bowel movements for which the options are 1–8. The PRO-UC Diary can be used to generate an overall summary score as well as a score for Mayo stool frequency and rectal bleeding. In contrast, the UC-PRO/SS provides a summary score for abdominal symptoms (based on 3 of the individual items) and a score for bowel signs and symptoms based on the other 6 items.

Similarly, there is broad overlap for symptoms of moderate-to-severe CD between the two CD tools. The PRO-CD Diary additionally includes bowel incontinence and general well-being, while the CD-PRO/SS additionally includes passing gas and bloating in belly [16]. Scores for individual items in the PRO-CD Diary are reported frequencies of symptoms, except for the assessment of abdominal pain (11-point scale), nausea severity (4-point scale), and general well-being (5-point scale). In contrast, all responses for the UC-PRO/SS are within a specified range of 0–4, except for the number of bowel movements for which the options are 1–8. The PRO-CD Diary can be used to generate an overall summary score from 8 items, excluding the 11-point abdominal pain score and 5-point general well-being score, which can be used to calculate the CDAI score. In contrast, the CD-PRO/SS provides a summary score for abdominal symptoms (based on 3 of the individual items) and a score for bowel signs and symptoms based on the other 6 items.

Differences in the items and scoring systems for the two sets of PROs may mean that a particular PRO is more appropriate according to the specific patient population being studied and/or the anticipated response to therapy. The minor differences in the symptoms included in the corresponding UC and CD tools may favor one or other tool in certain clinical situations. For instance, use of the PRO-CD Diary may be more appropriate than the CD-PRO/SS when effects on bowel incontinence are of interest, whereas effects on passing gas or bloating are better assessed using the CD-PRO/SS. Furthermore, where assessment of the frequency of symptoms is particularly relevant, the PRO-UC Diary and PRO-CD Diary tools are likely to be more sensitive than the UC-PRO/SS and CD-PRO/SS. Differences in the summary scores generated by the two sets of tools may also favor one or other tool in certain clinical settings.

Several limitations should be borne in mind when interpreting the findings of the research described herein. First, both studies involved only 12 participants. While the inclusion/exclusion criteria aimed to ensure that participants were representative of patients in whom the resulting tools would be used, the small number of participants and the fact that all were US residents and most were Caucasian mean they may not be fully representative of the overall IBD population intended to use these tools. Second, disease location was not recorded in the studies. Third, the final e-diary in each study was evaluated by only four participants. Finally, to achieve full validation of the PRO-UC and PRO-CD tools, psychometric assessment in an independent cohort is required; further, analysis of factorial validity, factor invariance and convergent/discriminant validity may be warranted. Despite these limitations, the results from our studies suggest that the PRO-UC Diary and the PRO-CD Diary are likely to be robust tools for the assessment of symptoms in patients with moderate-to-severe UC or CD.

## Conclusions

Both the PRO-UC Diary and the PRO-CD Diary were developed and validated in accordance with FDA recommendations for the rigorous development of disease-specific PRO tools, meaning that there are now two tools that can be used in clinical trials to assess response to treatment in patients with UC or CD. Psychometric analyses of the PRO-UC and PRO-CD Diaries are warranted to fully evaluate their properties and value for use in clinical trials. The availability of two tools for each disease, with differences in the aspects measured and their scoring systems, will provide clinicians with a choice of PRO tools for use appropriate to the setting. These tools should thus help further the development of better treatments for UC and CD that will improve management of symptoms and limit disease progression, both of which can have a major impact on patients’ lives.

## Supplementary Information

**Additional file 1.** COREQ (COnsolidated criteria for REporting Qualitative research) item checklist for the ulcerative colitis (UC) and Crohn’s disease (CD) qualitative studies.

## Data Availability

The data sets generated and/or analyzed during the current study are available from the corresponding author on reasonable request to researchers who provide a methodologically sound proposal. The data will be provided after their de-identification, in compliance with applicable privacy laws, data protection, and requirements for consent and anonymization.

## Abbreviations

CD: Crohn’s disease
CD-PRO/SS: Crohn’s Disease Patient-Reported Outcomes Signs and Symptoms
CDAI: Crohn’s Disease Activity Index
COREQ: COnsolidated criteria for REporting Qualitative research
EMA: European Medicines Agency
FDA: US Food and Drug Administration
IBD: Inflammatory bowel disease
IRB: Institutional review board
PRO: Patient-reported outcome
UC: Ulcerative colitis
UC-PRO/SS: Ulcerative Colitis Patient-Reported Outcomes Signs and Symptoms

## References

[1] Kobayashi T, Siegmund B, Le Berre C, Wei SC, Ferrante M, Shen B, Bernstein CN, Danese S, Peyrin-Biroulet L, Hibi T (2020). Ulcerative colitis. Nat Rev Dis Primers.

[2] Roda G, Chien Ng S, Kotze PG, Argollo M, Panaccione R, Spinelli A, Kaser A, Peyrin-Biroulet L, Danese S (2020). Crohn's disease. Nat Rev Dis Primers.

[3] Wallace KL, Zheng LB, Kanazawa Y, Shih DQ (2014). Immunopathology of inflammatory bowel disease. World J Gastroenterol.

[4] Fumery M, Singh S, Dulai PS, Gower-Rousseau C, Peyrin-Biroulet L, Sandborn WJ (2018). Natural history of adult ulcerative colitis in population-based cohorts: a systematic review. Clin Gastroenterol Hepatol.

[5] Feuerstein JD, Isaacs KL, Schneider Y, Siddique SM, Falck-Ytter Y, Singh S (2020). Committee AGAICG: AGA clinical practice guidelines on the management of moderate to severe ulcerative colitis. Gastroenterology.

[6] Sands BE (2004). From symptom to diagnosis: clinical distinctions among various forms of intestinal inflammation. Gastroenterology.

[7] Lichtenstein GR, Loftus EV, Isaacs KL, Regueiro MD, Gerson LB, Sands BE (2018). ACG clinical guideline: management of Crohn's disease in adults. Am J Gastroenterol.

[8] Armuzzi A, Liguori G (2021). Quality of life in patients with moderate to severe ulcerative colitis and the impact of treatment: a narrative review. Dig Liver Dis.

[9] Cohen RD (2002). The quality of life in patients with Crohn's disease. Aliment Pharmacol Ther.

[10] Armuzzi A, DiBonaventura MD, Tarallo M, Lucas J, Bluff D, Hoskin B, Bargo D, Cappelleri JC, Quirk D, Salese L (2020). Treatment patterns among patients with moderate-to-severe ulcerative colitis in the United States and Europe. PLoS ONE.

[11] Kalafateli M, Triantos C, Theocharis G, Giannakopoulou D, Koutroumpakis E, Chronis A, Sapountzis A, Margaritis V, Thomopoulos K, Nikolopoulou V (2013). Health-related quality of life in patients with inflammatory bowel disease: a single-center experience. Ann Gastroenterol.

[12] van der Have M, van der Aalst KS, Kaptein AA, Leenders M, Siersema PD, Oldenburg B, Fidder HH (2014). Determinants of health-related quality of life in Crohn's disease: a systematic review and meta-analysis. J Crohns Colitis.

[13] Reflection paper on the regulatory guidance for the use of health-related quality of life (HRQoL) measures in the evaluation of medicinal products. https://www.ema.europa.eu/en/regulatory-guidance-use-health-related-quality-life-hrql-measures-evaluation-medicinal-products.

[14] Guidance for industry—patient-reported outcome measures: use in medical product development to support labeling claims. https://www.fda.gov/media/77832/download. 10.1186/1477-7525-4-79 PMC1629006 17034633

[15] Higgins PDR, Harding G, Revicki DA, Globe G, Patrick DL, Fitzgerald K, Viswanathan H, Donelson SM, Ortmeier BG, Chen WH (2017). Development and validation of the ulcerative colitis patient-reported outcomes signs and symptoms (UC-pro/SS) diary. J Patient Rep Outcomes.

[16] Higgins PDR, Harding G, Leidy NK, DeBusk K, Patrick DL, Viswanathan HN, Fitzgerald K, Donelson SM, Cyrille M, Ortmeier BG (2017). Development and validation of the Crohn's disease patient-reported outcomes signs and symptoms (CD-PRO/SS) diary. J Patient Rep Outcomes.

[17] Flood E, Silberg DG, Romero B, Beusterien K, Erder MH, Cuffari C (2017). Development of the pediatric daily ulcerative colitis signs and symptoms scale (DUCS): qualitative research findings. BMC Res Notes.

[18] Higgins PD, Harding G, Patrick DL, Revicki DA, Globe G, Viswanathan HN, Trease S, Fitzgerald K, Borie DC, Leidy NK (2013). Development of the ulcerative colitis patient-reported outcomes (UC-PRO) questionnaire. Gastroenterology.

[19] Guideline on the development of new medicinal products for the treatment of ulcerative colitis. https://www.ema.europa.eu/en/documents/scientific-guideline/guideline-development-new-medicinal-products-treatment-ulcerative-colitis-revision-1_en.pdf.

[20] Ulcerative colitis: clinical trial endpoints guidance for industry. http://www.fda.gov/downloads/Drugs/GuidanceComplianceRegulatoryInformation/Guidances/UCM515143.pdf.

[21] Tong A, Sainsbury P, Craig J (2007). Consolidated criteria for reporting qualitative research (COREQ): a 32-item checklist for interviews and focus groups. Int J Qual Health Care.

[22] Joffe H, Yardley L, Marks DF, Yardley L (2004). Content and thematic analysis. Research methods for clinical and health psychology.

