# Supplementary material for: Development and content validation of patient-reported outcomes tools for ulcerative colitis and Crohn’s disease in adults with moderate-to-severe disease
Source: Health Qual Life Outcomes. 2022 May 7;20:75. doi: 10.1186/s12955-022-01975-1 (PMC9078017; doi:10.1186/s12955-022-01975-1)
Supplement: Supplementary file 1 — Additional file 1. COREQ (COnsolidated criteria for REporting Qualitative research) item checklist for the ulcerative colitis (UC) and Crohn’s disease (CD) qualitative studies. [file 12955_2022_1975_MOESM1_ESM.pdf]

Additional file 1 COREQ (COnsolidated criteria for REporting Qualitative research) item checklist for the ulcerative colitis (UC) and Crohn's disease (CD) qualitative studies

Topic	Item	Guide questions/description	Responses
**Domain 1: Research team and reflexivity**			
Personal characteristics			
Interviewer/facilitator	1	Which author/s conducted the interview or focus group?	Beverly Romero. Not all interviewers qualified as authors.
Credentials	2	What were the researcher's credentials? e.g. PhD, MD	MA.
Occupation	3	What was their occupation at the time of the study?	Senior Outcomes Researcher in Patient-Centered Outcomes at ICON plc.
Gender	4	Was the researcher male or female?	Female.
Experience and training	5	What experience or training did the researcher have?	Interviewers received study-specific training on the study procedures and the interview guide. The interviewers were also very experienced in conducting similar research and types of interviews.
Relationship with participants			

Relationship established	6	Was a relationship established prior to study commencement?	No relationship was established.
Participant knowledge of the interviewer	7	What did the participants know about the researcher? e.g. personal goals, reasons for doing the research	No information about the researcher regarding personal goals, biases, or reasons for undertaking the research were communicated to the participant.
Interviewer characteristics	8	What characteristics were reported about the interviewer/facilitator? e.g. bias, assumptions, reasons and interests in the research topic	No information about the interviewer/facilitator was reported.
**Domain 2: Study design**			
Theoretical framework			
Methodological orientation and theory	9	What methodological orientation was stated to underpin the study? e.g. grounded theory, discourse analysis, ethnography, phenomenology, content analysis	Interview data were analyzed using a combination of thematic and content analysis.
Participant selection			
Sampling	10	How were participants selected? e.g. purposive, convenience, consecutive, snowball	Participants were recruited through a US market research recruitment agency (Global Perspectives). Convenience sampling was used; participants had shown prior interest in taking part in similar research.

Method of approach	11	How were participants approached? e.g. face-to-face, telephone, mail, email	Potential participants were identified from the market research recruitment agency, Global Perspectives, database (consisting of patients with a wide variety of medical conditions who had all given prior permission to be contacted for research purposes). Those meeting inclusion criteria were approached to discuss the study, allowing participants to make an informed decision before consenting to participate.
Sample size	12	How many participants were in the study?	24 (UC: 12; CD: 12).
Non-participation	13	How many people refused to participate or dropped out? Reasons?	Not applicable.
Setting			
Setting of data collection	14	Where was the data collected? e.g. home, clinic, workplace	The first two rounds of participant interviews were conducted over the telephone using paper-based administration of the tool. The third round was delivered in an electronic format, the e-diary, and was assessed in face-to-face interviews at an interview facility.
Presence of non-participants	15	Was anyone else present besides the participants and researchers?	No.

Description of sample	16	What are the important characteristics of the sample? e.g. demographic data, date	Participants were aged 31–59 years in the UC study and 18–61 years in the CD study, and 75% of participants in both studies were in remission at the time of the interview. In both groups, 10 participants (83%) and 2 participants (17%) were receiving anti-tumor necrosis factor therapy and anti-integrin therapy, respectively. Five participants (42%) and 2 participants (17%) in the UC and CD groups, respectively, were receiving 5-aminosalicylic acid treatment. Five participants (42%) in each study reported comorbidities.
Data collection			
Interview guide	17	Were questions, prompts, guides provided by the authors? Was it pilot tested?	Interviews were conducted using a semi-structured interview script. The interview script was not pilot-tested with patients, though revisions were made as needed based on feedback between rounds of patients.
Repeat interviews	18	Were repeat interviews carried out? If yes, how many?	No.
Audio/visual recording	19	Did the research use audio or visual recording to collect the data?	All interviews were audio-recorded.
Field notes	20	Were field notes made during and/or after the interview or focus group?	Audio-recordings of each interview were transcribed and de-identified.

Duration	21	What was the duration of the interviews or focus group?	Interviews lasted approximately 60–90 minutes.
Data saturation	22	Was data saturation discussed?	Concept saturation was assessed based on the number of interviews taken to identify the symptoms.
Transcripts returned	23	Were transcripts returned to participants for comment and/or correction?	No.
**Domain 3: Analysis and findings**			
Data analysis			
Number of data coders	24	How many data coders coded the data?	Data were coded by three researchers.
Description of the coding tree	25	Did authors provide a description of the coding tree?	A description of the coding tree was not described in the manuscript. A coding framework was developed initially using the interview and was adjusted as themes emerged from the data.
Derivation of themes	26	Were themes identified in advance or derived from the data?	The interview guide was used as a starting point to develop the coding framework and the coding framework was adjusted as themes emerged from the data, using a thematic analysis approach.
Software	27	What software, if applicable, was used to manage the data?	MaxQDA, v11.

Participant checking	28	Did participants provide feedback on the findings?	No.
Reporting			
Quotations presented	29	Were participant quotations presented to illustrate the themes/findings? Was each quotation identified? e.g. participant number	Yes, quotes from participants were captured. However, such quotations are not presented in this manuscript and are therefore not identified.
Data and findings consistent	30	Was there consistency between the data presented and the findings?	Yes.
Clarity of major themes	31	Were major themes clearly presented in the findings?	Yes.
Clarity of minor themes	32	Is there a description of diverse cases or discussion of minor themes?	Limited.

Developed from: Tong A, Sainsbury P, Craig J. Consolidated criteria for reporting qualitative research (COREQ): a 32-item checklist for interviews and focus groups. Int J Qual Health Care. 2007;19(6):349–57.
